# Myocardial Ischemia in Patients with COVID-19 Infection: Between Pathophysiological Mechanisms and Electrocardiographic Findings

**DOI:** 10.3390/life12071015

**Published:** 2022-07-08

**Authors:** Ștefania Teodora Duca, Adriana Chetran, Radu Ștefan Miftode, Ovidiu Mitu, Alexandru Dan Costache, Ana Nicolae, Dan Iliescu-Halițchi, Codruța-Olimpiada Halițchi-Iliescu, Florin Mitu, Irina Iuliana Costache

**Affiliations:** 1 Department of Internal Medicine I, Faculty of Medicine, University of Medicine and Pharmacy “Grigore T. Popa”, 700145 Iasi, Romania; stefania-teodora.duca@email.umfiasi.ro (Ș.T.D.); radu-stefan.miftode@umfiasi.ro (R.Ș.M.); ovidiu.mitu@umfiasi.ro (O.M.); dan-alexandru.costache@umfiasi.ro (A.D.C.); ana_nicolae@umfiasi.ro (A.N.); halitchi.iliescu@umfiasi.ro (D.I.-H.); florin.mitu@umfiasi.ro (F.M.); irina.costache@umfiasi.ro (I.I.C.); 2 Department of Cardiology, “St. Spiridon” Emergency County Hospital, 700111 Iasi, Romania; 3 Department of Cardiovascular Rehabilitation, Clinical Rehabilitation Hospital, 700661 Iasi, Romania; 4 Department of Cardiology, Arcadia Hospital, 700620 Iasi, Romania; 5 Department of Mother and Child Medicine-Pediatrics, University of Medicine and Pharmacy “Grigore T. Popa”, 700115 Iasi, Romania; olimpiada.iliescu@umfiasi.ro; 6 Department of Pedriatics, Arcadia Hospital, 700620 Iasi, Romania

**Keywords:** COVID-19, myocardial ischemia, electrocardiography

## Abstract

Given the possible pathophysiological links between myocardial ischemia and SARS-CoV-2 infection, several studies have focused attention on acute coronary syndromes in order to improve patients’ morbidity and mortality. Understanding the pathophysiological aspects of myocardial ischemia in patients infected with SARS-CoV-2 can open a broad perspective on the proper management for each patient. The electrocardiogram (ECG) remains the easiest assessment of cardiac involvement in COVID-19 patients, due to its non-invasive profile, accessibility, low cost, and lack of radiation. The ECG changes provide insight into the patient’s prognosis, indicating either the worsening of an underlying cardiac illnesses or the acute direct injury by the virus. This indicates that the ECG is an important prognostic tool that can affect the outcome of COVID-19 patients, which important to correlate its aspects with the clinical characteristics and patient’s medical history. The ECG changes in myocardial ischemia include a broad spectrum in patients with COVID-19 with different cases reported of ST-segment elevation, ST-segment depression, and T wave inversion, which are associated with severe COVID-19 disease.

## 1. Introduction

In the last month of 2019, an outbreak of atypical respiratory disease occurred in China, in the city of Wuhan. Shortly after the first cases, it was discovered that a novel coronavirus was responsible for the next pandemic, as the World Health Organization declared. Since it was structurally related to the virus that caused acute respiratory distress syndrome during 2002–2003, the novel coronavirus was named as the severe acute respiratory syndrome coronavirus-2 (SARS-CoV-2) and the outbreak was called Coronavirus disease 19 (COVID-19) [1,2].

The most common presentation of COVID-19 is pneumonia, but different reports describe different complications, from thromboembolism, myocarditis, and acute coronary syndrome to multiple organ failure [3,4,5]. Because of the possible pathophysiological links between myocardial ischemia and the SARS-CoV-2 infection, several studies have focused on them in order to improve patients’ morbidity and mortality [3,6]. As acute coronary syndromes have been reported to be up to 30%, they may have a significant role in worsening clinical outcomes in patients with COVID-19 [6,7]. Moreover, long COVID-19 syndrome is characterized by persistent tissue damage and inflammation following an acute bout of SARS-CoV-2 infection, mainly manifested through chronic fatigue and dyspnea, but also, among others, with cardiac symptoms. The inflammation of the heart tissues, especially myocardial inflammation which is as prevalent as 78% in post-COVID-19 patients, leads to tachycardia, palpitations, and chest pain in the months post-discharge [8,9].

A series of case reports published have shown that patients following acute COVID-19 infections have developed postural tachycardia syndrome (POTS). This involves an increase in over 30 beats per minute in the heart rate in adults or over 40 beats per minute in patients between the ages of 12–19 in the first 10 min since standing in an orthostatic position, associated with orthostatic intolerance, in the absence of orthostatic hypotension, for a minimum of 3 months [10].

Since the epidemic of COVID-19, multiple studies of hypercoagulability have attracted attention; the pathophysiology aspects of myocardial ischemia have been studied in detail and we focused on electrocardiographic changes in infected individuals [11].

The electrocardiogram (ECG) remains the most facile assessment method for cardiac involvement in COVID-19 patients, due to its non-invasiveness, accessibility, low cost, and radiation-free investigative profile [12,13,14]. Although the management during a patient’s admission is safer when laboratory values and imaging become available, ECG can be quickly performed without exposing a large number of people to SARS-CoV-2 [12,15].

Therefore, we propose a review of different publications regarding the ECG findings in patients with COVID-19 and myocardial ischemia and the pathophysiological pathways behind them. The ECG pattern can predict the severity of the COVID-19 infection and clinical outcomes such as mortality, as several studies have shown [11,16,17,18]. In the COVID-19 era, patients with chest pain require more attention and high clinical suspicion. ECG interpretation is more challenging, as it can also show chronic abnormalities of an underlying cardiac disease [19,20].

The ECG changes in myocardial ischemia include a broad spectrum in patients with COVID-19, different cases of ST-segment elevation, ST-segment depression, and T-wave inversion being reported, which were associated with a severe form of COVID-19 disease [21,22]. Several authors have described P-wave changes, in the context of atrial infarction, which is supplied in 60% of cases by the right coronary artery and in 40% by the circumflex artery through the superior ramus ostium cava [23].

We searched PubMed and Google Scholar for articles published from December 2019 to March 2022 and compiled a database using the keywords “COVID-19”, “SARS-CoV-2”, “coronavirus”, “ECG”, “electrocardiography”, “myocardial ischemia”, “myocardial infarction”, “acute coronary syndrome”, “hypercoagulability”, “pathophysiology” and all their combinations.

The results included original papers, retrospective studies, prospective studies, reports, systematic reviews, meta-analyses, and case reports, focusing on COVID-19 and ECG changes in myocardial ischemia. We reviewed relevant articles for data to comprehensively discuss and describe the pathophysiological mechanisms and ECG changes in patients with COVID-19 and acute coronary syndrome.

## 2. Pathophysiology of Myocardial Ischemia in COVID-19 Patients

In the recent COVID-19 pandemic, there were challenges on several levels, from the difficulties of controlling the spread of the disease, to the complexity of diagnosing and treating the multisystemic complications of this infection. Even though it is primarily an infectious disease, the impact of COVID-19 on the cardiovascular system is significant, with complications such as pericarditis, myocarditis, pulmonary embolism, myocardial infarction, and exacerbation of chronic heart failure being described more often as the pandemic progressed [24,25].

Acute and chronic coronary diseases, already some of the most prevalent diseases in the general population, have played a major role in the years of COVID-19 infection as factors of severity and mortality [25]. The problems related to coronary heart disease in the context of COVID-19 infection were multiple: late patient presentation to the hospital, misleading symptoms by the interference of the two diseases, reduced intensive care unit (ICU) capacity, delayed treatment by COVID-19 testing and protocols, hs-troponin values altered by lung infection, and severe prognosis due to coronary heart disease and associated COVID-19 infection. All these issues challenge the diagnostic and therapeutic approach of coronary artery diseases, which reaches beyond guidelines and requires new studies on the particular mechanisms of myocardial ischemia in the context of COVID-19 infection [26,27,28].

Typical symptoms of coronary disease (chest pain, fatigue, and dyspnea) have lost their specificity and sensitivity as COVID-19 infection often has similar manifestations which delays the diagnosis [29]. Cardiac troponin, a cornerstone biomarker in the diagnosis of myocardial infarction, had elevated values above the 99th percentile in 8–62% of patients with COVID-19 infection, with the highest incidence and mortality recorded in patients with severe infection [30]. In addition to cardiac causes for high troponin values (acute coronary syndrome, myocarditis, and Takotsubo syndrome), several noncardiac conditions, such as sepsis, critical illness, and pulmonary embolism, can lead to increased troponin [30]. Therefore, ECG changes are critical in establishing the diagnosis of coronary diseases. Particular aspects of ECG tracing in patients with COVID-19 have also been described and should be recognized for the proper management of these cases [29,30].

Among coronavirus patients, a substantial number developed myocardial injury or acute coronary syndrome, complications more prevalent if the patient had a previous cardiovascular disease, worsening his prognosis and mortality [31,32,33].

For a better understanding of the diverse mechanisms involved in the ECG ischemia changes in patients with COVID-19, it is important to make the distinction between two main entities: acute cardiac injury (ACI) and acute coronary syndrome (ACS). ACI is defined as the elevation of high-sensitivity cardiac troponin (hs-cTn) above the 99th percentile of its upper limit of normal, usually with stable and unchanging values, or evidence of new electrocardiographic (ECG) or echocardiographic abnormalities, and may have ischemic or non-ischemic etiology [34].

The causes for ACI may involve an ACS (type I myocardial infarction-due to plaque rupture or thrombosis, type II myocardial infarction-due to oxygen supply/demand mismatch, or myocardial injury due to disseminated intravascular coagulation) or a non-ischemic etiology as: myocarditis, Takotsubo cardiomyopathy (stress-induced cardiomyopathy), acute pulmonary embolism, cytokine release syndrome, and direct viral myocardial invasion. The differential diagnosis between an ischemic and a non-ischemic cause for the ACI may need additional imaging tests (CT Coronary Angiography (CTCA), lung CT scan, or cardiac magnetic resonance (CMR)), guided also by serial hs-cTn testing. It was noted that patients with ACI caused by an ACS have dynamically changing values of hs-cTn, with a rise and fall, as opposed to other types of ACI where troponin had steadily increasing values [34,35].

According to European Society of Cardiology (ESC) guidelines, ACS defines the clinical situations of acute myocardial ischemia or infarction, which can be further classified in three types: unstable angina, Non-ST-elevation myocardial infarction (NSTEMI), and ST-elevation MI (STEMI) [36]. Acute myocardial infarction (AMI) is defined by consistent myocardial ischemia that leads to cardiomyocyte necrosis. The diagnosis involves the detection of dynamic changes in hs-cTn associated with at least one of the following: symptoms of myocardial ischemia, new ischemic ECG changes, development of pathological Q waves on ECG, imaging evidence of viable myocardium loss or new regional wall motion abnormality in a pattern consistent with an ischemic etiology, or intracoronary thrombus detected on angiography or autopsy [36].

The presence of ACS has been reported in a significant number of patients with COVID-19 infection, which has had a negative impact on the prognosis [31,32]. For these patients, some particularities for ACS were noticed such as higher incidence of stent thrombosis, multiple thrombotic culprit lesions, high thrombus burden, and angiographic evidence of non-obstructed coronary arteries, which raised concerns about the diverse mechanisms involved [37,38]. In addition to the usual cardiovascular risk factors, to date, the main mechanisms involved in the development of ACS in COVID-19 patients are: prothrombotic activation of the coagulation cascade, endothelial dysfunction, cytokine-mediated systemic inflammatory response, and hypoxic injury due to oxygen supply/demand imbalance [39].

The effects of COVID-19 infection on the coagulation cascade are not yet fully understood, involving either direct viral aggression or an exacerbated cytokine-mediated inflammatory response [40]. The presence of hemostatic abnormalities was suggested in these patients by higher D-dimer values, decreased platelets count, prolonged prothrombin time, and increased levels of fibrinogen degradation products [41,42]. These abnormalities have been reported in severe cases of COVID-19 pneumonia and are associated with a negative prognosis [41,42,43,44]. The prothrombotic state is maintained by the overexpression of ultra-large von Willebrand factor multimers and tissue factor, induced by the cytokines produced during the systemic inflammatory response, which triggers the activation of the coagulation cascade [45]. Another possible mechanism for SARS-CoV-2-related coagulopathy is the presence of lupus anticoagulant antibodies, that may be induced in any infectious or inflammatory disease. The exposed phospholipids of the endothelium to the immune system leads to thrombus formation [46].

Endothelial cells have the ability to respond to many humoral and hemodynamic stimuli by producing regulatory molecules that are part of the defense mechanism of vascular homeostasis. The endothelium plays a significant role in controlling the immune response by promoting leukocyte migration into extravascular spaces, which helps fight infections and promotes tissue repair [47]. In response to inflammatory cytokines such as interleukin IL-1, IL-6, and tumor necrosis factor-α (TNF-α), endothelial cells exhibit adhesion molecules on their surface (e.g., E-selectin, P-selectin, intercellular adhesion molecule-1, vascular cell adhesion molecule-1 (VCAM-1), and integrins), which increases leukocyte binding [48]. Accumulation of inflammatory cells causes endothelial dysfunction through reduced NO bioavailability and increased oxidative stress via activation of NADPH oxidase [49]. The studies confirm the role of adhesion molecules in COVID-19 complications, indicating that high circulating levels of VCAM-1 and E-selectin are associated with increased COVID-19 severity, as a meta-analysis from 2021 showed [50]. Furthermore, in critically ill patients with COVID-19 a high expression of inflammatory cytokines, supporting the role of the cytokine release storm in severe cases has been identified [51].

Endothelial dysfunction is the main cause for coronary artery disease in patients with traditional cardiovascular risk factors (diabetes, smoking, hypertension, and older age) [52]. As the thrombotic complications in patients with COVID-19 increase, endothelial injury is incriminated, either by a direct viral effect or by the action of inflammatory cytokines [53,54]. A direct effect of SARS-CoV-2 on the vascular endothelial glycocalyx (VEGLX) is suspected [55]. Glycocalyx damage usually is associated with pathological situations as inflammatory response, hyperglycemia, hypoxia, and ischemia/reperfusion injury, which may also be present in COVID-19 pneumonia [55,56,57,58]. The theory is supported by the measurements of circulating levels of VEGLX components in COVID-19 patients, where higher concentrations were detected for VEGLX injury biomarkers such as syndecan-1, hyaluronic acid, and sTie-2 [27,59]. In the presence of an inflammatory state, the endothelium loses its antithrombotic, anticoagulant, and profibrinolytic capacity as a result of tissue factor expression, von Willebrand factor release, thromboxane production, and plasminogen activator inhibitor-1 (PAI-1) production [60]. Moreover, the inflammatory cytokines act on the endothelial cells to generate superoxide anions, with enhanced local oxidative stress [61]. Overproduction of endothelin-1 contributes, as well, to the endothelial imbalance, acting as a prothrombotic agent and vasoconstrictor [62].

**Figure 1:**
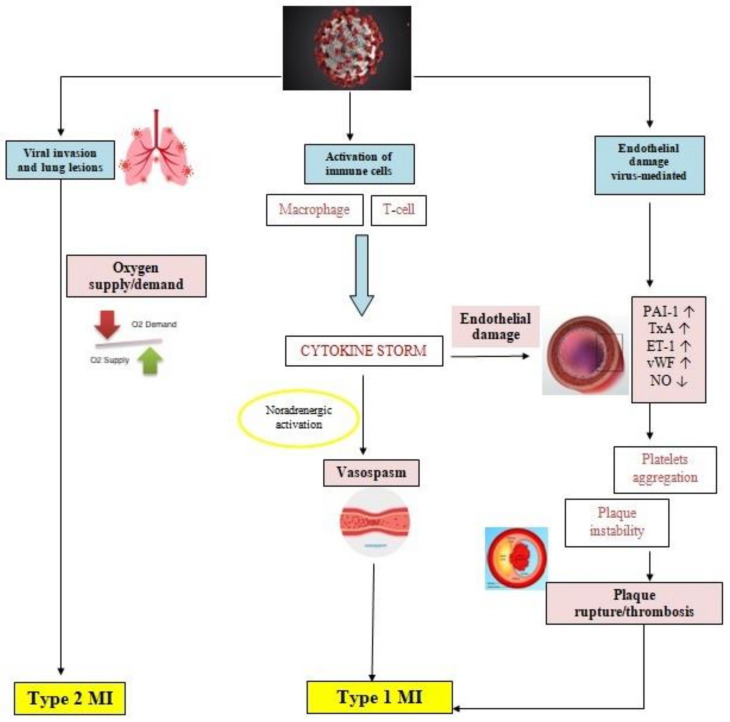
Pathophysiology of myocardial ischemia in COVID-19 patients. COVID-19 infection acts by biding to the ACE2 receptors present on the surface of the host cell, which may be pneumocytes, macrophages, or endothelial cells. Pulmonary infection may range from mild disease to pneumonia and ARDS in severe forms, which in cases of severe respiratory impairment causes hypoxia and due to an oxygen supply/demand mismatch, a type 2 MI. An aberrant inflammatory response is typically described in COVID-19 infection, with the release of cytokines and molecules involved in inflammation, such as IL-1, IL-6, IL-7, TNFα, and IFNγ. The negative effects of cytokines manifest by increasing the production of oxidative stress agents and prothrombotic factors, which damage the endothelial function. Furthermore, SARS-CoV-2 may interact directly with the molecules expressed on the surface of the endothelial cells. The inflammatory environment promotes platelets activation and aggregation, upregulates the sympathetic nervous system, increasing the risk of instability of preexisting atheromatous plaques and coronary spasm. All these mechanisms predispose to plaque rupture and thrombosis, leading to type 1 MI. ACS: acute coronary syndrome; ACE2: angiotensin-converting enzyme 2; COVID-19: coronavirus disease 2019; IFNγ: interferon γ; IL-1: interleukin 1; IL-6: interleukin 6; IL-7: interleukin 7; MI: myocardial infarction; SARS-CoV-2: severe acute respiratory syndrome coronavirus 2; TNFα: tumor necrosis factor α.

Atherosclerotic disease progression is enhanced by the presence of inflammatory response, which maintains a prothrombotic state and promotes vasoconstriction through sympathetic activity [63,64]. The disturbed balance between prothrombotic and antithrombotic factors promotes erosion or rupture of the atherosclerotic plaque, resulting in coronary thrombosis and ACS [65]. In COVID-19 infection, an exaggerated immune response and a cytokine chain reaction, known as cytokine storm (CS), has been observed [40]. The CS starts with the production of interleukin-1 (IL-1), which has some particularities: it may induce its own gene expression, but also promotes the production of other inflammatory factors such as tumor necrosis factor-alpha (TNFα), interleukin-6 (IL-6), and chemoattractant molecules [66]. All these factors create a prothrombotic and antifibrinolytic imbalance, generating thrombosis and local tissue injury [67]. In SARS-CoV-2 patients, increased levels of inflammatory mediators, part of the CS, such as IL-1, IL-6, IL-10, IFN, granulocyte colony stimulating factor (G-CSF), monocyte chemoattractant protein (MCP1), macrophage inflammatory protein 1 alpha (MIP1A), platelet-derived growth factor (PDGF), TNFα, and vascular endothelial growth factor (VEGF) were detected, which supports the contribution of CS to the development of ACS (Figure 1) [44,68,69].

Prothrombotic activation of the coagulation cascade, endothelial dysfunction, and cytokine-mediated systemic inflammatory response are mechanisms that involve the presence and progression of atherosclerotic lesions, that become unstable and clinically manifest as a type I myocardial infarction (MI). However, a significant number of COVID-19 patients showed ECG signs of ischemia in the absence of atherothrombotic lesions [70,71,72]. In these patients a type II MI is suspected, due to a mismatch between oxygen supply and demand. The severe hypoxic state, tachyarrhythmias, anemia, sepsis, hypotension, and shock may induce myocardial damage and have often been described in critically ill patients with COVID-19 [73]. Due to the severe situations that induce type II MI, the prognosis is worse, with a higher rate of in-hospital mortality compared with type I MI [74].

Cases of myocardial infarction with nonobstructive coronary arteries (MINOCA) were reported in some case series of STEMI and COVID-19 patients [75,76]. The possible mechanisms involved include coronary vasospasm, microthrombi, and plaque erosion, triggered by the exaggerated inflammatory response, oxidative stress and endothelial dysfunction [76,77,78]. The limited access to more advanced techniques as intravascular imaging, pharmacological provocation test, and cardiac magnetic resonance, in COVID-19 patients, constitutes an obstacle to a complete understanding of the underlying mechanisms [78,79].

Troponin is the gold standard biomarker used for detection of myocardial ischemia, as reflects the presence of cardiomyocyte damage. In ACS, either STEMI or NSTEMI, the dynamic of circulating troponin levels is similar, regardless of the ECG pattern (ST-elevation, ST-depression, or inverted T wave), the main factor influencing its value is the moment of detection after symptom onset [80]. Typically, troponin cannot be detected in the first 1–2 h of myocardial necrosis, but only approximately 2–4 h after the onset of myocardial injury. Thereby, in cases where patients seek early medical advice, the troponin levels may be normal or slightly increased, which is an indication for repeating the troponin measurements. Serum levels can persist being elevated for up to 4–7 days for troponin I, and 10–14 days for troponin T [81]. We did not find studies indicating correlations between troponin levels and particular ECG aspects of myocardial ischemia or infarction. However, a certain type of ACS that should be mentioned is Wellens syndrome which, despite its great severity and high risk, manifests frequently with troponin within normal limits, which can be falsely reassuring [82]. In COVID-19 patients, troponin levels have often been elevated as a result of cardiac injury, with or without AMI, which highlights the important role of ECG in establishing the diagnosis [83]. However, troponin proved to be valuable in anticipating the prognosis of COVID-19 patients, as Santoso et al. presented in a meta-analysis, which concluded that troponin levels were associated with higher mortality, need for care in the intensive care unit (ICU) and a more severe form of COVID-19 infection [84]. The hypothesis is also confirmed by a study that investigated the risk of mortality in patients with COVID-19, new T-wave inversions (TWI) and troponin levels. Jorge Romero et al. showed in their study that patients with elevated troponin and TWI had an 80% mortality risk, compared with a 35% mortality risk when isolated TWI with normal values of troponin were present [85].

## 3. Electrocardiographic Changes in Myocardial Ischemia in COVID-19 Patients

### 3.1. ST-T Abnormalities

ST-T abnormalities are well-known and described in patients with myocardial ischemia and they include ST-elevation or depression, T-wave inversion, and nonspecific ST-T-wave changes. While acute subendocardial ischemia causes ST-segment depression, acute transmural ischemia causes ST-segment elevation, due to the electrical repolarization currents responsible for the ST-segment, which are deviated towards the inner layer of the heart in the first case and towards the outer layer of the heart in the latter. Even though the ECG signs of an acute ST-elevation are more accurate than an acute non-ST-elevation, the repolarization abnormalities of a myocardial infarction can persist indefinitely [86,87].

Patients infected with SARS-CoV-2 who associate myocardial injury may show ST-segment elevation, ST-segment depression, or T-wave inversion on ECG [18,19]. Howbeit, the ST-segment elevation in COVID-19 patients requires differential diagnosis with pericarditis and myocarditis, while myocardial ischemia may be due to both obstructive coronary artery diseases and a mismatch between oxygen supply and demand [88]; these findings have been observed more frequently in severe patients [13,22]. Among the critically ill patients, an abnormal ECG with ST-T changes reaches a frequency of 48.5%, while in patients with a severe type of COVID-19, 25.7% have this abnormality according to Wang et al. [89]. Most studies found that ST-T abnormalities were the most common changes, occurring in up to 40% of patients from different hospitals, including Wuhan Asia General Hospital [15,19,88,90]. T-wave inversion was the most common repolarization change in several studies, including the one of Galidevara et al., who reported that 27.7% of ECG changes were due to this abnormality [7,22]. However, while Rosen et al. reported in his study that 21% patients had an ECG with ST-T modification, Poterucha et al. reported that 10% of the presentations had this abnormality, T-wave inversion was observed in up to 29% of this group, which is similar to other studies. This may be due to the number of patients included in the studies, the second one having a number of patients seven times higher [14,91].

Regarding the localization of the repolarization abnormality, lateral and antero-lateral changes on the ECG were the most frequently described, being also associated with worse clinical outcomes [92,93,94].

The ST-T modifications on the ECG can be used as an indicator for poor prognosis, including more frequent need for mechanical ventilatory support, increased need for ICU admission, and finally increased mortality [13,19,89]. Patients with ST-T changes on the admission ECG are more likely to show progression towards a severe form, being associated with the severity of the COVID-19 infection and a worse prognosis [13,22,95]. Therefore, the COVID-19 patients detected with myocardial ischemia on the ECG should be monitored for sudden cardiac death [13].

Patients with ST-T abnormalities had elevated cardiac biomarkers, such as troponin, and more frequent requirement for vasoactive treatment [14,96,97]. Although mild troponin elevation was a frequent finding in the study of Chorin et al., which is often associated with non-vascular etiologies, biomarkers of cardiac damage should alert clinicians of a poor prognosis [97].

#### 3.1.1. STEMI Pattern

The ST-segment elevation is sometimes associated with a fatal evolution or a worse prognosis in patients, especially in those with COVID-19 infection. Several studies attest the presence of an ST-segment elevation, characteristic for myocardial infarction, in patients with SARS-CoV-2 infection. Therefore, it is important, especially in patients with COVID-19, to differentiate the ischemic ST-elevation from the one in a possible myopericarditis [11,76,98].

The ST-segment elevation myocardial infarction (STEMI) is defined as new ST-elevation at the J point in two or more contiguous leads, ≥0.25 mV in men below the age of 40 years, ≥0.2 mV in men over the age of 40 years, or ≥0.15 mV in women in leads V2–V3, and/or ≥0.1 mV in other leads [86,99,100].

A multicenter case series study from six hospitals in New York, which included patients with ST-segment elevation and COVID-19, showed that in 44% of patients a diagnosis of myocardial infarction was established, whereas 56% had a non-coronary myocardial injury. Half of the patients underwent invasive intervention with angiography, but only two-thirds of them had obstructive disease. This finding suggests that a more prevalent COVID-19 was associated with a non-obstructive type of MI, probably determined by type 2 myocardial infarction [11,76,98]. However, univariate analyses showed that ST-segment elevation had a strong correlation with patients’ mortality, being an independent prognostic factor. Sonzor et al. demonstrated in their article in American Journal of The Medical Science that ST-segment elevation can be associated with clinical outcomes in hospitalized patients with COVID-19 [101]. Moreover, the occurrence of ST-segment elevation during hospitalization is also an alarm sign for a poor prognosis, this manifestation being due to the side effects of therapeutic agents, the direct attack of the virus on myocardial tissue, or an indicator of myocardial ischemia [102].

Mccullough et al. reported that only 0.7% of the presentations had localized ST-segment elevation on the ECG, their data suggesting that this was not a common finding in the New York Presbyterian Hospital in the first months of the pandemic [15]. These data were sustained by another study which showed that localized ST-segment elevation was observed in only 0.5% of the presentations during the same months [97].

There was no specific localization of the myocardial infarction in COVID-19 patients as several studies have shown, some concluding that inferior myocardial infarction was the most frequent, while others showing that anterior myocardial injury was the most common type [75,94,100]. An important aspect to mention is that STEMI represented the first clinical manifestation of COVID-19 for most patients with myocardial infarction and only a few developed ST-segment elevation during hospitalization for COVID-19 [75]. Even though we need to be aware of the atypical clinical presentation of COVID-19 with cardiovascular manifestations, physicians should not misdiagnose STEMI even after admission, especially if the patient has chest pain [3,103]. We researched almost all published cases of STEMI and we found an approximately equal localization of the myocardial infarction, with no statistical significance between the affected walls: inferior myocardial infarction-5 cases, inferolateral myocardial infarction-4 cases, anterior myocardial infarction-5 cases, or anterolateral myocardial infarction-3 cases. Interestingly, patients who had a subacute myocardial infarction, with ST-segment elevation and Q waves, had an anterior STEMI with late presentation [3,11,103,104,105,106,107,108,109,110,111]. Patients with COVID-19 and STEMI who were admitted to the hospital had several cardiovascular risk factors with a past medical history of type 2 diabetes mellitus, hypertension, hyperlipidemia, or overweight body mass index (BMI) [98,104]. This suggests that cardiovascular risk factors in patients with COVID-19 infection play an important role, as this association leads to a more prevalent occurrence of a myocardial infarction. The mean age of the total cases of STEMI associated with COVID-19 infection found in literature was 54.4 years of age, and the majority of patients were male, only four being female with acute STEMI [3,11,103,104,105,106,107,108,109,110,111]. However, it was observed that the youngest patients with ST-segment elevation myocardial infarction and COVID-19 were female [109,110]. Different authors published articles which included patients with STEMI, but without COVID-19 and concluded that the prognostic for those patients was better, not only because they did not have SARS-CoV-2 infection, but also because the angiography was performed faster [104].

We also found studies with cases of transient ST-segment elevation on the ECG, the possible explanations being a type 2 myocardial infarction due to inflammatory activation, respiratory failure, and severe hypoxia [97,112]. MINOCA due to type 2 acute myocardial infarction causes the appearance of deep Q waves on the subsequent electrocardiograms [113]. Stefanini et al. showed in their study that in approximately 40% of patients with COVID-19 and STEMI, a culprit lesion was not identifiable in the angiography [75]. Therefore, it is not necessary to perform an angiography in all patients with STEMI and COVID-19, but we need to place in balance the risks and benefits and to understand the pathophysiological mechanisms behind de ECG aspects [104,108].

#### 3.1.2. ST-Depression Pattern

ST-segment depression is defined as a new horizontal or down-sloping ST-depression of at least 0.05 mV in two contiguous leads [87,99]. The non-ST-elevation myocardial infarction (NSTEMI) is seen on ECG as ST-segment depression, T-wave inversion or nonspecific ST-segment, and T-wave changes [86]. The variety of ECG findings requires a detailed analysis and raises differential diagnosis problems, as ST-T abnormalities were the most reported ECG findings in patients with COVID-19 infection [88,89,90].

Although various studies have not shown any cases of ST-segment elevation, they have found ST-segment depression, reported in approximately 5.3% of cases, this manifestation also being associated with a high mortality [6,20,114]. As we described above, MINOCA can be challenging due to the atypical clinical presentation, especially in patients with NSTEMI [6]. Moreover, ST-segment depression related to MINOCA was associated with high levels of cardiac biomarkers [101]. Antwi-Amoabeng et al. showed an incidence of 8.6% of ST-segment depression, with a significant association between this ECG finding and troponin levels [115]. Due to the more frequent cases of ST-segment depression compared with ST-segment elevation, we did not find in literature specific cases of NSTEMI, but only studies on large cohorts [6,114,116].

#### 3.1.3. T-Wave Inversion and Other Patterns of ST-Abnormalities

T waves are normally inverted in leads III, aVR, and V1. T-wave inversions (TWI) produced by myocardial ischemia are classically narrow, symmetric, and have variable depth. Moreover, they have mirror patterns, start in the second part of the repolarization, and may be accompanied by a positive or negative U wave [99,115].

Heberto et al. showed that ischemic T-wave inversion was the most frequent electrocardiographic finding in COVID-19 patients and observed a relationship with mortality in these patients [117]. Other studies demonstrated that T-wave abnormalities were present in up to 49% of the COVID-19 patients [118,119]. Almost all studies showed that T-wave inversion appeared more frequently in the cardiac injury group and in patients over 74 years of age [111,120]. However, Capaccione et al. reported a case of a 36-year-old patient with myocardial ischemia and inferior T-wave inversion, confirming the hypothesis of various studies showing that patients with COVID-19 infection have an increased risk of developing severe cardiovascular disease at a younger age [121]. The number of leads with T-wave inversion pattern was significantly correlated with the elevation of cardiac injury biomarkers, such as troponin [119]. Both of these findings were associated with increased mortality and the need for intubation. Romero et al. conducted a study on 3225 patients with COVID-19 infection, where T-wave inversion was observed in 6% of patients, with the most frequent localization (71%) in the lateral leads (DI, aVL, V5–V6) [18]. Therefore, T-wave inversion remains the most frequent repolarization abnormality and is associated with poor outcomes and death, especially in those with elevated troponin levels [14,121]. The issue for patients with T-wave inversions due to NSTEMI is that during the COVID-19 era, even if the patients were classified as high-risk NSTEMI, angiography was not performed, and conservative treatment during isolation was preferred due to the high risk of infection. However, this strategy may contribute to increasing mortality, as Suryawan et al. reported in their case presentation [122,123].

### 3.2. Q Waves

Pathological Q waves are defined as the occurrence of Q waves in at least two contiguous leads as follows: any Q wave in leads V2–V3 of at least 0.02 s or QS complex in leads V2 and V3; Q wave of at least 0.03 s and at least 0.1 mV deep or QS complex in leads I, II, aVL, aVF, or V4–V6; R wave of at least 0.04 s in V1–V2 and R/S of at least one with a concordant positive [86,99].

We found in literature some cases of subacute myocardial infarction in COVID-19 patients, with Q wave being observed on ECG in approximately 4% of cases. The location was in both the anterior and inferior territories, and the patients maintained the ST-segment elevation on ECG [20,92]. Because the Q wave remains the only sign of myocardial infarction on ECG, a remote myocardial infarction is more difficult to recognize. Moroni et al. presented three cases of myocardial infarction with late presentation, due to the patients’ fear of being infected with COVID-19 in the hospital, who treated themselves at home and were hospitalized long after a good therapeutical procedure could have been performed (Table 1) [105]. This is an alarm signal for patient educational programs, as the SARS-CoV-2 infection alone does not have a poor prognosis, but it can exacerbate preexisting conditions [92,105].

### 3.3. Specific Electrocardiographic Patterns

Cases of COVID-19 patients who had specific electrocardiographic patterns were described in literature.

#### 3.3.1. Takotsubo Pattern

Takotsubo syndrome (TTS) was first described in the 1990s, as an acute myocardial infarction, but with normal angiography and patients recovering within days or weeks. Even though it was initially considered a benign disease, subsequent studies have demonstrated a higher mortality rate than in the normal population. The most common finding on ECG is the ST-segment elevation in the precordial leads, with less common ST-segment depression or abnormal Q waves than for myocardial infarction and with transient changes [124,125,126,127].

TTS occurs in 90% of cases in women, often being preceded by emotional stress, therefore the COVID-19 pandemic can be considered a triggering factor [128]. The pathophysiology is associated with high plasma levels of catecholamines, the impact of adrenergic activity on cardiac myocytes causing ascending ST-segment elevation and J point depression. Moreover, high catecholamine levels might increase oxygen demand, inducing vasospasm and myocardial injury, patients with SARS-CoV-2 infection being exposed to a high endogenous secretion as a compensatory intravenous infusion, which are used as a therapeutic method [23,129]. Another determinant mechanism involves the hypothalamic-pituitary-adrenal (HPA) axis, which is activated in COVID-19; several studies have shown that cortisol and adrenocorticotrophic hormones were dysregulated. As the levels of cortisol are significantly higher in COVID-19 patients and the HPA axis induces catecholamine secretion, a relationship between TTS and hypercortisolemia was described. An important aspect to mention is the BNP/troponin ratio used to differentiate TTS from acute myocardial infarction, as TTS is associated with higher brain natriuretic peptide (BNP) and lower troponin levels [129].

Literature presents many cases of TTS in patients with COVID-19, the most specific ECG abnormalities being ST-segment elevation in the anterior leads [11]. Approximately 2–4% of patients with COVID-19 had TTS, with a higher prevalence in critically ill patients, as some studies have reported. Although TTS predominates in women, about 30% of the patients with COVID-19 and TTS are males [129]. The first typical case of TTS with apical ballooning observed on echocardiography in a patient with COVID-19 was reported by Meyer et al. Both emotional and physical stress by the pandemic were considered trigger factors [130].

ST-segment elevation in leads I and aVL and diffuse ST-T-wave changes were found in two female patients with COVID-19, who were subsequently diagnosed with TTS because the cardiac catheterization revealed normal coronary arteries. One of them even admitted to feeling anxious by the reports and images of the COVID-19 pandemic, TTS being an indirect outcome of quarantine-induced stress [128,129,130].

Although it was considered a benign disease, TTS patients with COVID-19 have a higher mortality rate than patients who have only TTS [131,132]. Furthermore, Barbieri et al. showed that in a hospital from Lombardy, during the COVID-19 pandemic, more patients with TTS have been diagnosed with TTS than in the same months of the previous years. This is an additional argument for the impact of COVID-19 on stress-induced cardiomyopathy [133].

#### 3.3.2. Wellens Pattern

**Figure 2:**
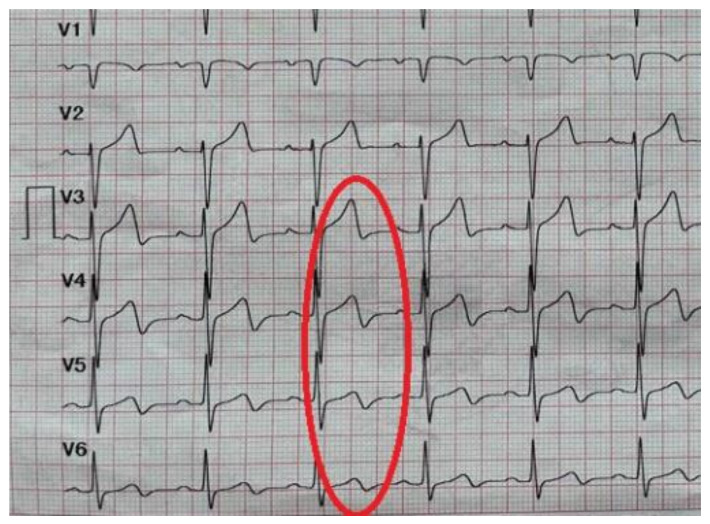
Wellens pattern: deeply biphasic T waves in leads V3–V6 (The collection of the “St. Spiridon” Hospital’s Cardiology Clinic, Iasi, Romania).

Wellens syndrome is a specific disease for critical stenosis of the proximal left anterior descending coronary artery. The ECG shows deeply inverted or biphasic T waves in leads V2–V3, often in leads V1 and V4 and occasionally in leads V5–V6 (Figure 2). Patients with Wellens syndrome are at risk of a large anterior wall myocardial infarction, even though they are pain-free and the cardiac enzymes are normal [82,134,135].

We found in literature four cases of Wellens syndrome in patients with SARS-CoV-2 infection, three of them were male and one female (Table 2). The mean age was 73 years old, significatively higher than the group with ST-segment elevation. On the ECG, all patients had biphasic T waves in V2–V3, three of them with additionally negative T waves in leads V4–V6. Only one patient had typical chest pain, three patients had only dyspnea. The female patient did not have an angiography performed, in one patient an emergency angiography was performed and the other two patients had a coronary angiography after a few days of medical treatment. All patients were treated with drugs for NSTEMI, with a high-intensity statin, dual antiplatelet aspirin, P2Y12 inhibitors, and anticoagulant [135,136]. Regarding the therapeutic approach, we observed a difference during the pandemic. If the first published case of Wellens syndrome did not receive an angiography because of the COVID-19 status and the risk of infection, Caiati et al., the authors who published the latest case of Wellens syndrome during the COVID-19 pandemic, chose another therapeutic approach and the patient underwent an angiography [136,137,138,139]. This may be due to the fact that at the beginning of the pandemic, information about COVID-19 was scarce, a conservatory treatment was preferred in order to keep as little physical contact as possible. Even though for the diagnosis of Wellens syndrome physicians need to perform an angiography, during the pandemic, the European Society of Cardiology and the American College of Cardiology recommended medical management during the acute phase [136].

In addition to the ECG aspect, patients should be investigated for cardiovascular risk factors, as well as a structural evaluation with echocardiography, coronary angiography being the investigation that assists the ECG findings [136]. However, because the high risk of infection during the COVID-19 pandemic required new diagnostic methods, Caiati et al. proposed a non-invasive way that can assess the obstructive atherosclerosis through transthoracic enhanced Doppler echocardiography (E-Doppler TTE). This allows the identification of the coronary blood flow velocity, even though this investigation has never been tested in a Wellens syndrome. The authors used it for the first time the E-Doppler TTE in an acute syndrome and this can open new perspectives on the management of an acute coronary syndrome [139].

#### 3.3.3. De Winter Pattern

The De Winter ECG pattern shows an upsloping ST-segment depression at the J-point in leads V1–V6, followed by peaked symmetrical T waves, being associated with left anterior descending artery occlusion, with a positive predictive value of 95% [140,141,142].

**Figure 3:**
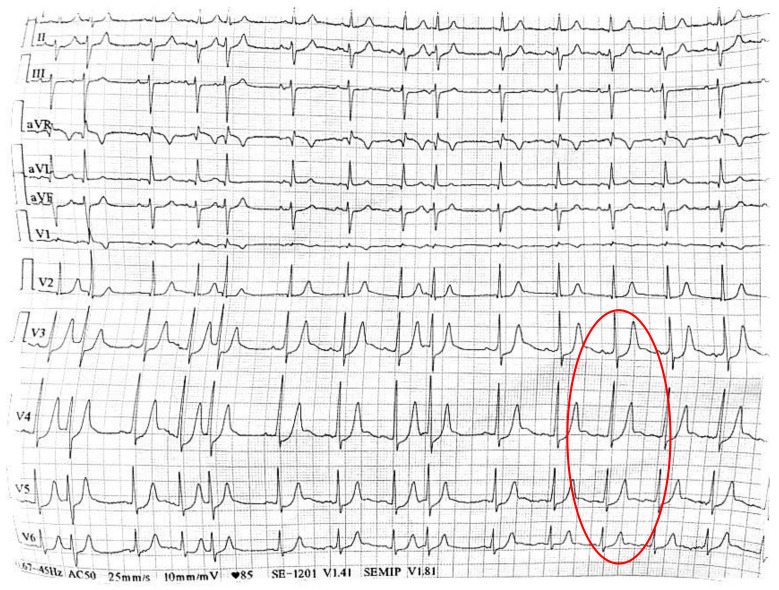
De Winter pattern: upsloping ST-segment depression at the J-point, followed by peaked symmetrical T waves in lead V3, lead V4, lead V5 and lead V6 (The collection of the “St. Spiridon” Hospital’s Cardiology Clinic, Iasi, Romania).

Almendro-Delia et al. published the only case of De Winter syndrome associated with COVID-19 infection, which occurred in a 33-year-old male patient. Due to his infectious disease, atypical chest pain suggestive for pericarditis, and the ECG aspect with ST-segment depression in V1–V6 followed by tall T waves, the first diagnosis was acute pericarditis (Figure 3). The laboratory result which revealed high troponin levels and the echocardiographic aspect with apical and antero-lateral hypokinesis with an apical thrombus at this region, led to the reinterpretation of the ECG as a De Winter aspect. The angiography confirmed the thrombotic occlusion of the proximal left anterior descending artery, the outcome being favorable. Therefore, in the COVID-19 era, it is necessary to make a detailed differential diagnosis, even in apparently young healthy patients. The ECG aspects can be misinterpreted as early repolarization or pericarditis, delaying proper reperfusion treatment. Thus, the De Winter pattern is the equivalent of an early STEMI, an aspect which should not be ignored [143].

#### 3.3.4. Triangular Electrocardiographic Pattern

Another unique ECG presentation of STEMI is represented by the triangular QRS-ST-T waveform, also known as the “shark fin pattern”, which is defined as a giant wave resulting from the fusion of the QRS complex, the ST-segment and the T wave [76,144]. This uncommon ECG pattern reflects the left main coronary artery involvement, with a large area of transmural myocardial ischemia and poor in-hospital prognosis [76].

**Figure 4:**
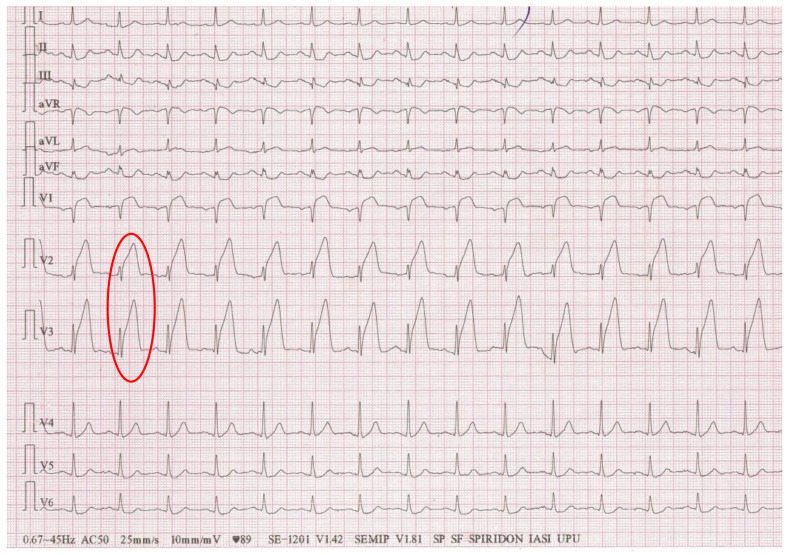
Triangular ECG pattern in leads V2 and V3: fusion of the QRS complex, the ST-segment and the T wave (The collection of the “St. Spiridon” Hospital’s Cardiology Clinic, Iasi, Romania).

A 32-year-old female patient with COVID-19 infection and shortness of breath, showed a “shark fin pattern” in leads I, II, III, and aVL post-cardiac arrest (Figure 4). Although an angiography was necessary, given her critically ill clinical status and the echocardiographic aspect with mid-wall hypokinesis suggestive for Takotsubo, the intervention was postponed. The “shark fin pattern” is a rare ECG finding, typical for left main coronary artery, but because of the patient’s past history of intravenous drug abuse and hepatitis C, over which the COVID-19 infection overlapped, this ECG aspect can be determined by several other factors, such as abnormal laboratory tests [145].

### 3.4. Other Electrocardiographic Aspects Associated with Myocardial Ischemia in COVID-19 Patients

Even though new left bundle branch block, right bundle branch block, or poor R-wave progression were described as different changes suggestive for myocardial ischemia, in COVID-19 patients we did not find specific publications or studies regarding only the incidence of these aspects in infected patients [14,93].

## 4. Why Is It Important to Analyze Myocardial Ischemic-like Electrocardiographic Changes in COVID-19 Patients?

The ECG findings described above are important for differential diagnosis, especially with myopericarditis. In patients with COVID-19 myocarditis, approximately 50% had ST-segment elevation on the ECG, the distinction from myocardial infarction being difficult, although ST-segment elevation in myocarditis is diffuse, not focal as in acute coronary syndrome [18,90]. Moreover, in myopericarditis, ST-segment is characterized by an elevation of the J point with a concave shape, while in STEMI the elevation of the J point has a convex shape. Additional tests are required, troponin being mildly elevated in myopericarditis and the cardiac ultrasound showing increased wall thickness, decreased ejection fraction, or global hypokinesis [18,124]. Finally, late gadolinium enhancement on MRI can reveal the final diagnosis of myocarditis [18]. We found in literature some cases of patients with COVID-19 infection and ST-T abnormalities seen on ECG being interpreted as an acute coronary syndrome with an MRI performed after the angiography showing normal coronary arteries, and thus establishing the diagnosis myopericarditis [11].

Another differential diagnosis to consider is a fever-induced Brugada pattern, as COVID-19 is frequently associated with fever [75].

The management of patients with myocardial infarction suspicion has also suffered changes in the COVID-19 era. Given the difficult differential diagnosis with myocarditis, it is important for the reperfusion therapy not to be postponed in the emergency department. Moreover, in COVID-19 patients with a strong suspicion for NSTEMI, initial medical therapy is the optimal option. Finally, SARS-CoV-2 rapid testing remains mandatory even in the emergency setting and protocols are continuously being adapted [146].

## 5. Conclusions

Understanding the pathophysiological aspects of myocardial ischemia in patients infected with SARS-CoV-2 can open a broad perspective on the proper management of each patient. The ECG changes provide insight into the patient’s prognosis. Although we found the same ECG aspects for myocardial ischemia as before the pandemic, these are much more severe, occur in younger and healthier patients, and the stress-induced cardiomyopathy is much more common in COVID-19 patients. Further studies are needed to elucidate the particular mechanisms of myocardial ischemia in patients infected with COVID-19 in order to promote more specific treatments and to prevent these complications.

## Figures and Tables

**Table 1:** Patients with subacute myocardial infarction (adapted after Moroni et al., 2020).

Age	Sex	Symptoms	Time to in-Hospital Presentation	Treatment at Home	ECG Aspect
64	M ^1^	Chest pressure Shortness of breath	10 days	Homemade natural remedies	Q waves and ST-segment elevation on the anterior leads
65	F ^1^	Epigastric tightness Dyspnea Orthopnea	5 days	Antiacids	Q waves and ST-segment elevation on the anterior leads
60	M ^1^	Hypotension Diaphoresis Respiratory distress	4 days	None	Q waves and ST-segment elevation on the anterior leads

^1^ M: male; F: female.

**Table 2:** Patients with Wellens syndrome and COVID-19 infection.

Article	Age	Sex	Symptoms	ECG Findings	Treatment	Angiography
Prousi et al.	75	F ^1^	Fatigue Dyspnea	Diffuse T-wave inversions in precordial leads Biphasic T waves in V1–V2	Statin Aspirin P2Y12 inhibitors Heparin	Not performed
Elkholy et al.	86	M ^1^	Dyspnea	Biphasic T wave in V2–V3 T-wave inversion in V4–V6	Statin Aspirin P2Y12 inhibitors Enoxaparin	Chronic total occlusion of the right coronary artery. Severe disease of the first diagonal. Severe stenosis of the distal obtuse marginal 1
Di Spigno et al.	62	M ^1^	Atypical chest pain Dyspnea	Biphasic T waves in V2	-	Subocclusion of the proximal left anterior descending artery
Caiati et al.	69	M ^1^	Typical chest pain Dyspnea	T-waves inversion in V2–V3 T-wave flattening in V4–V6	Statin Aspirin P2Y12 inhibitors Heparin	Subocclusive stenosis of the proximal LAD

^1^ M: male; F: female.
